# Non‐visual cues and indirect strategies that enable discrimination of asymmetric mates

**DOI:** 10.1002/ece3.8790

**Published:** 2022-04-01

**Authors:** Roshan Kumar Vijendravarma, Pierre Leopold

**Affiliations:** ^1^ Institut Curie – Centre de Recherche, Genetics and Developmental Biology Unit INSERM U934 / CNRS UMR3215 Paris France

**Keywords:** developmental instability, extended phenotype, fluctuating asymmetry, mate choice, mate choice copying, multimodal courtship, non‐visual cues, sexual selection

## Abstract

The postulates of developmental instability–sexual selection hypothesis is intensely debated among evolutionary biologists, wherein despite a large amount of empirical data, evidence for or against it has been largely inconclusive. A key assumption of this hypothesis is that animals assess symmetry in potential mates as an indicator of genetic quality (developmental stability), and consequently use this information to discriminate against those with higher asymmetries while choosing mates. However, the perceptional basis that must underlie such discriminatory behavior (is symmetry a signal or is symmetry signaled) is not clearly defined. It is also argued that since asymmetry levels in natural populations are very low, the low signal‐to‐noise ratio would make accurate assessment of symmetry both difficult and costly. Rather than attempting to validate this hypothesis or even as to whether animals assess mate symmetry, this review simply aims to examine the plausibility that animals perceive symmetry (directly or indirectly) and consequently discriminate against asymmetric mates in response to perceived irregularities during courtship. For this, we review mate choice and courtship literature to identify potential sensory cues that might advertise asymmetry or lead to discrimination of asymmetric individuals. Although signaling associated with mate choice is commonly multimodal, previous studies on asymmetry have mainly focused on visual perception. In the light of a recent study (Vijendravarma et al., 2022, *Proceedings of the National Academy of Sciences of the United States of America*, 119, e2116136119), this review attempts to balance this bias by emphasizing on non‐visual perception of asymmetry. In conclusion, we discuss the methodological challenges associated with testing the role of multimodal cues in detecting mate asymmetry, and highlight the importance of considering ecological, behavioral, and evolutionary aspects of animals while interpreting empirical data that test such hypothesis.

## Graphical abstract

We review how multimodal non‐visual cues can signal morphological asymmetry of potential mates during courtship in diverse species across taxa. We also discuss how extended phenotypes and mate choice copying aid females to indirectly assess and discriminate against asymmetric males. With the current emphasis on multimodal communication during courtship, this review is well timed to rekindle interest in the role of symmetry during mate choice.

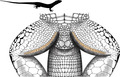

## 1 INTRODUCTION

The ‘decision’ of whom to mate with has far‐reaching evolutionary consequences through sexual selection, and has intrigued biologists for decades (Andersson & Iwasa, 1996; Hare & Simmons, 2019). Mate choice‐associated fitness not only directs animal decisions that favor evolution of secondary sexual traits and courtship displays but also facilitates reproductive isolation that can lead to evolutionary diversification and speciation (Andersson & Iwasa, 1996). Across taxa, courtship displays present signals and cues that help individuals reliably predict the species, reproductive status, and quality of potential mates (Candolin, 2003; Mitoyen et al., 2019). It is increasingly being recognized that both males and females can assess and choose their potential mates, suggesting that sexual selection can simultaneously operate on both sexes (Caro et al., 2021). However, traditionally in most species, females (and males in sex role reversed species) are often choosier owing to their larger investment in reproduction, both in terms of physiology ‘gamete production’ and behavior ‘nursing and parental care’ (Hare & Simmons, 2019). Alongside the quality of the sexual traits that males exhibit (Mitoyen et al., 2019), females also prefer larger males during courtship (Andersson & Iwasa, 1996), but only when body size signals increase competitive ability, reproduction, and survival (Fairbairn et al., 2007). Larger males are maybe less preferred when mating success decreases due to reduced agility (Zhu et al., 2016) or when size‐assorted mating occurs (Andersson & Iwasa, 1996).

The developmental instability–sexual selection hypothesis (DI‐SS) was conceptualized in 1980s; it proposes that females can assess fluctuating asymmetry (FA), the random deviations from perfect bilateral symmetry in sexual traits, as a reliable indicator of phenotypic (and perhaps genotypic) quality (developmental stability) of a potential mate (Moller & Pomiankowski, 1993). Although intensely debated and extensively tested empirically, the data supporting this hypothesis are heterogeneous (Moller & Pomiankowski, 1993; Polak, 2003, 2008; Polak & Taylor, 2007). While some reviews tried to consolidate this inconclusive debate through meta‐analysis detected publication bias (Palmer, 1999), others who documented its history explain how interest in this hypothesis waned after accusations of scientific misconduct and fraud (Debat, 2016; Houle, 1998; Palmer, 2000). Former studies in this field across taxa are also biased toward investigating visual assessment of male FA and we argue that this could be one of the factors contributing toward the observed heterogeneity. Support for this idea comes from our recent study that reports how *Drosophila melanogaster* females discriminate asymmetrically winged males during courtship based on the asymmetric songs (auditory cues) they produced (Vijendravarma et al., 2022). Rather than validating this hypothesis, this review aims to examine mate choice literature for potential non‐visual cues and other indirect methods that females could utilize to either detect FA in courting males (directly or indirectly) or discriminate against asymmetric individuals.

Similar to our recent finding, we found several cases wherein it appears that symmetry could be signaled through non‐visual cues during courtship. However, we acknowledge that in most of these cases it has been neither proved that asymmetry is being assessed by an individual nor that it operates as a signal. Nevertheless, we review these examples as a first step to highlight the potential role of non‐visual cues in FA‐mediated sexual selection to motivate further research.

To survive, animals constantly assess costs and benefits of their potential decisions by detecting and processing signals/cues in their complex and unpredictable environment. Such signals/cues are multimodal and often processed through several sensory modalities simultaneously, to make decisions linked to behaviors like foraging, predation, and mating (Halfwerk et al., 2019; Kulahci et al., 2008; Weissburg et al., 2014). Multimodality during mate choice is no exception, where females rely not only on anticipated sexual signals but also on inadvertent cues detected during courtship (Candolin, 2003). The DI‐SS hypothesis assumes that females perceive and assess asymmetry in males and alter their responses accordingly. The negative correlations observed between male FA and female choice in empirical studies are attributed to cue reading and signaling. However, in several empirical studies where asymmetric males were less preferred, it remains unclear whether asymmetry itself was assessed by females, or whether their choice was based on other traits that correlated with asymmetry. In other words, since developmental instability can affect bilateral symmetry as well as myriad of other quality‐related traits, testing whether asymmetry in a focal trait alone has affected female mate choice has been difficult (Uetz & Taylor, 2003). However, such challenges can be overcome by improvising experimental designs, for example, using genetic tools to induce organ‐specific asymmetry (Vijendravarma et al., 2022).

Former reviews on FA‐based sexual selection are biased toward visual perception (Swaddle, 1999, 2000), possibly reflecting our own sensory preference for visual cues. Convincingly, several examples of vision‐mediated female preference for males with symmetrical markings are known: vertical bars in swordtail fishes (*Xiphophorus cortezi*) (Morris & Casey, 1998); spots in guppies (*Poecilia reticulata*) (Sheridan & Pomiankowski, 1997); and chest plumage pattern in zebra finches (*Taeniopygia guttata*) (Swaddle & Cuthill, 1994). Nevertheless, visual cues can also be dynamic; for example, in wolf spiders (*Schizocosa ocreata*), females reject males with asymmetric bristle tufts on their forelegs that are displayed during leg tapping (Uetz & Smith, 1999). However, given the multimodal nature of sexual communication, we argue that symmetry could also be perceived or detected through other signaling modes, operating independently or combinatorially (Uetz & Taylor, 2003; Vijendravarma et al., 2022).

Perception of FA through both visual and non‐visual cues was previously reviewed, posing interesting questions on how developmental instability affects animal communication (Uetz & Taylor, 2003). Following which, interest in understanding FA‐mediated sexual selection declined for reasons discussed earlier, hindering any scientific advances in this field (Debat, 2016; Palmer, 1999). This and our recent work (Vijendravarma et al., 2022) inspired us to write this review.

## 2 NON‐VISUAL PERCEPTION OF ASYMMETRY

Although some studies in humans and other animals seem to predict mating decisions based on bilateral symmetry, preference for symmetrical mates might not always reflect preference or assessment of symmetry per se (Uetz & Taylor, 2003). Nonetheless, studies on FA‐mediated sexual selection commonly presume that mate symmetry is assessed visually (Swaddle, 1999), ignoring that it could also be inferred through cues perceived via other sensory modalities. This seems counterintuitive since it is evident across taxa that mate choice is mediated by multimodal courtship displays (Candolin, 2003; Hare & Simmons, 2019; Mitoyen et al., 2019) delicately orchestrated through concomitant signals that besides vision, targets other sensory modalities like chemosensory, mechanosensory, and auditory. Furthermore, vision is not indispensable for mate choice in animals that are nocturnal, blind, or dwell in dark habitats (caves, food grains, underground, and within plants); visual traits are seldom useful. Nocturnal bats use echolocation for mate choice, wherein female preference for larger males is mediated by higher echolocation peak frequency (Puechmaille et al., 2014). In comparison to surface‐dwelling fish that visually choose larger mates, cave‐dwelling conspecifics exploit non‐visual sensory cues to maintain the same mating preference (Plath et al., 2003, 2005). Below, we review non‐visual cues that individuals exploit directly or indirectly to infer mate symmetry while courting. In addition, we also include mate choice studies where non‐visual cues could possibly signal asymmetry, but needs to be tested explicitly.

### 2.1 Chemosensory

Animals use semiochemicals in sexual communication to track, attract, locate, and assess mate quality (Johansson & Jones, 2007), operating both over long distances through smell (olfaction) and perceived upon contact through taste (gustatory). As humans, visual detection of asymmetry by animals is easier to comprehend, but imagining it in the context of other sensory modalities, like chemical communication, is demanding (Meredith, 2001). Hence, visualizing semiochemicals as colored particles is possibly useful: which if volatile is ejected from the source, suspended in air as plumes, and settles on sensory organs of the receiver (that are often bilaterally structured, e.g., insect antennae and vertebrate nostrils); and if non‐volatile is present on surfaces and is picked up by sensory organs present on appendages upon contact. Since sex pheromones are secreted by bilaterally positioned glands or by organs along the body's midline, their distribution (quantity) in the ensuing plumes (for volatiles) and on surfaces (for non‐volatiles) is likely to be partitioned bilaterally too. Such visualization allows us to understand how differences in bilateral pheromone distribution might signal asymmetry. Animal navigation and chemical communication from within these plumes have been well investigated among insects while designing pheromone traps in the field of pest management (Murlis et al., 1992). Interestingly, studies analyzing spatial and temporal distribution of tracer concentrations within plumes show that the relative direction of the plume centerline and the consequent bilateral comparison of tracer concentrations is plausible (Takasaki et al., 2012; Webster et al., 2001). This suggests that whenever species‐specific orientation behavior and timed release of pheromone bursts during courtship are coupled, such bilateral assessment is plausible. Thus, increased FA in pheromone secretion system could signal asymmetry through quantitative or qualitative deviations of compounds in the pheromone cocktail that is emitted or secreted by a potential mate (Martin & Lopez, 2006).

**FIGURE 1:**
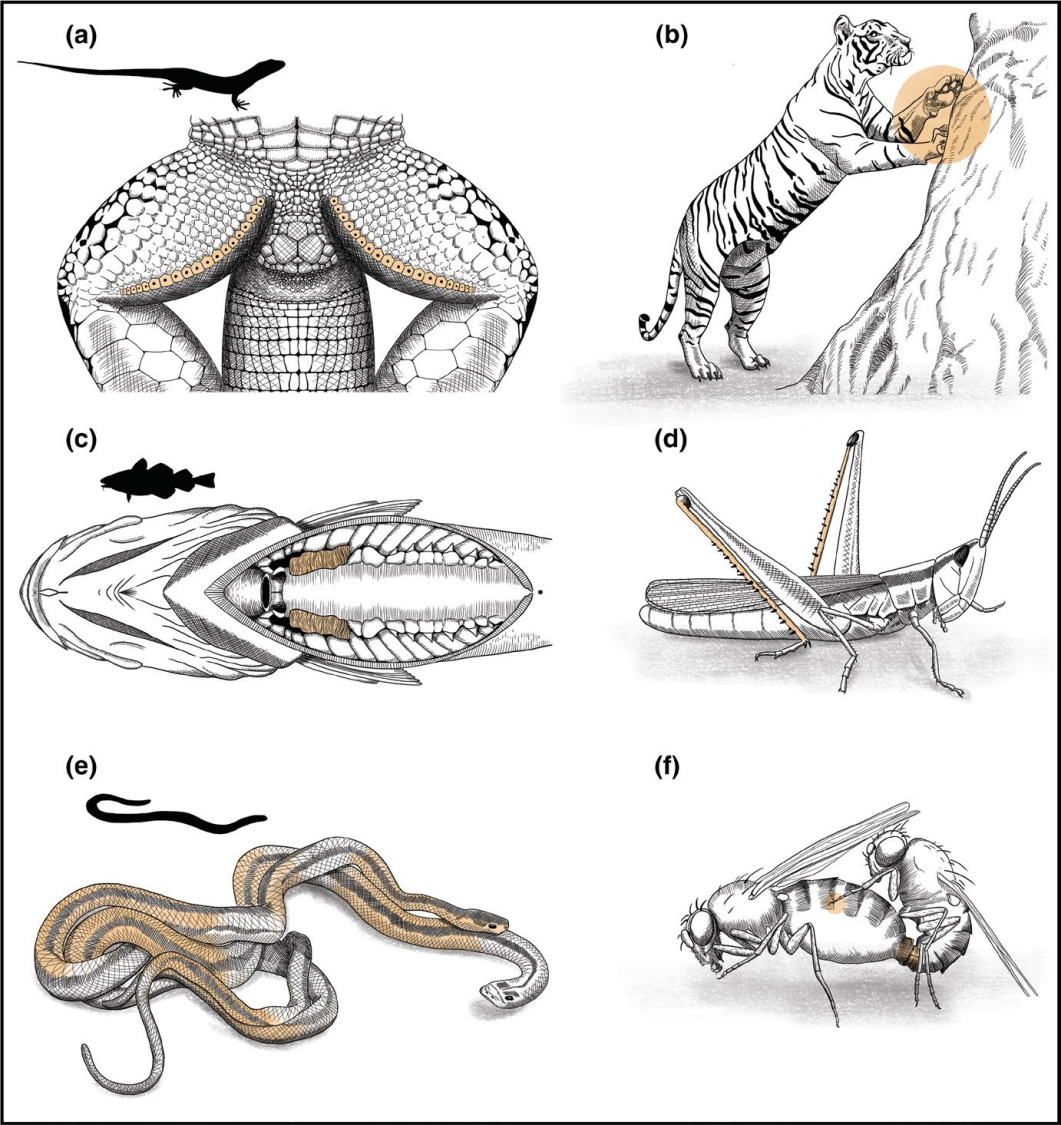
Non‐visual sexual communication (*highlighted*) associated with mate choice: (a, b) chemosensory: bilaterally located femoral gland pores of male Iberian lizard (a) and male royal Bengal tiger scent marking by scratching a tree trunk (b); (c, d) auditory: bilateral drumming muscles associated with the swim bladder in Atlantic cod (c) and slant faced male grasshopper with stiff spines on the hind legs used for stridulation (d); (e, f) mechanosensory: red garter snake male aligning alongside a female to copulate (e), and male and female fruit flies exchanging chemical, vibratory, and tactile stimuli during copulation (f)

Whether smell or taste of semiochemicals can reflect male asymmetry has been rarely tested. Female Iberian rock lizards (*Lacerta monticola)* preferentially associate with femoral gland secretions of males with low FA in their femoral pores while courting (Martin & Lopez, 2000, 2006) (Figure 1a). Interestingly, this preference is mediated by the female ability to discriminate higher levels of cholesta‐5,7‐dien‐3‐ol and ergosterol in the femoral secretions of symmetric males (Martin & Lopez, 2006). In Japanese scorpionfly (*Panorpa japonica*), females prefer to mate with males with relatively low FA in forewing length (Thornhill, 1992). This female mating preference was mediated by the male pair formation pheromone produced in the male eversible genital pouch, which when blocked with glue or wax abolished female preference for symmetry (Thornhill, 1992). In humans, a similar preference for body odor of males with symmetrical facial features has also been reported among women in their fertile phase of the ovulatory cycle (Gangestad & Thornhill, 1998; Rikowski & Grammer, 1999; Thornhill & Gangestad, 1999), but there is insufficient information on the underlying mechanisms. These studies propose that asymmetry in focal traits might be signaled through correlated changes in pheromone concentration or its distribution, but can nevertheless also reflect changes in age, size, diet, development, and health of the emitter. Alternatively, FA in pheromone secreting or distributing organs may vary the bilateral pheromone concentration, providing a direct cue to assess the degree of asymmetry.

Literature on animal courtship presents several other examples where bilaterality of semiochemicals possibly directs mate choice, and they appear to be more pronounced in nocturnal and territorial species. Males of greater sac‐winged bats (*Saccopteryx bilineata*) perform a ‘perfume blending’ behavior wherein their bilateral wing sacs are filled with bodily fluids and gland secretions for use during courtship (Voigt & Helversen, 1999). Interestingly, symmetric males in this species gain higher reproductive success, possibly through female choice (Voigt et al., 2004). Such bilateral perfuming is also exhibited by other bat species, like ‘dorsal patch’ in long‐nosed bats (*Leptonycteris curasoae*) (Muñoz‐Romo & Kunz, 2009) and ‘forearm crust’ in fringe‐lipped bats (*Trachops cirrhosus*) (Flores & Page, 2017). Terrestrial mammals similarly exhibit several scent‐marking behaviors, like scraping, claw raking, spraying (squirting urine and scent), and cheek/head/body rubbing, to signal mate availability and quality (Gosling & Roberts, 2001; Johnson, 1973; Soso et al., 2014), but behaviors plausibly also reflect asymmetry of the marking individual. Claw raking felids (cats) alternate their fore paws to scratch surfaces wherein scent glands in each foot could leave bilaterally distinguishable chemosensory cues (Figure 1b; reviewed in Harmsen et al. (2010)). Analogues function for other sexual secretions in mammals can be speculated: Temporal gland secretions in bull elephants during ‘Musth’ (Chelliah & Sukumar, 2013; LaDue et al., 2021); apocrine gland fields on wrists of lemurs, used in shoulder‐rubbing and wrist‐marking behaviors (Charpentier et al., 2008); anal gland and sac secretions in bears (Sergiel et al., 2017); and facial gland secretions of chiropteran species (Rehorek et al., 2010). These cases hint toward the innumerable examples that one could consider in other taxa (reptiles, amphibians, fishes, and insects) that have evolved higher orders of chemosensory communication (Houck, 2009; Johansson & Jones, 2007).

Sexual pheromones, how and what they communicate, their perception, the neuronal circuitries involved, and how they modulate sexual behaviors and mate choice, all these topics have received meticulous attention over the last few decades, from both the sender and the receiver perspective using empirically pliable and genetically trackable model systems spanning across taxa (Blum, 1996; Brezolin et al., 2018; Mucignat‐Caretta, 2014; Soso et al., 2014). We believe that the field is thus conducive to test if indeed asymmetry during mate choice can be signaled through chemosensory cues.

### 2.2 Auditory

Certain groups of animals like mammals, birds, insects, anurans, and fishes have evolved to utilize vocal or vibration‐generated sounds during their sexual communication. The male acoustic calls produced while courting are energetically costly and are thus considered to reliably signal male quality (Prestwich, 1994), communicating the calling male's species, body size, dominance status, and parasite load (Redpath et al., 2000). Interestingly, since these calls are produced by bilaterally placed sound‐producing organs (vocal cords, syrinx, vibrating wings, or special stridulating structures), asymmetry in these organs or in any structures that facilitate production of the sound could thus plausibly influence the quality of acoustic signal produced.

We recently detected such an effect of morphological asymmetry on male courtship song in the model organism *Drosophila melanogaster*. Male *Drosophila* sing species‐specific courtship song in the presence of a female by vibrating their unilaterally extended wing, while alternating between wings on both sides. We demonstrated that females rejected males with asymmetric wings during mate choice assays based on acoustic cues. To understand the effect of wing asymmetry on the courtship song, we made audiovisual recording of the courtship songs of symmetric and asymmetric males, and segmented the song into right and left wing‐generated bouts. Comparing auditory features of these side‐specific bouts revealed asymmetry in the songs produced by the rejected males with asymmetric wings (Vijendravarma et al., 2022). Although *Drosophila* courtship song has been investigated extensively since its discovery in 1962 (Shorey, 1962), this bilaterality had been ignored. Interestingly, another recent study has established that the preferred singing location for males is situated on either side of the female midline at angular positions behind her, and for best acoustic simulation of the female antenna, the male vibrates his right wing when positioned to the right of the female and vice versa (Morley et al., 2018). Together, these studies provide clear evidence for bilaterality in acoustic communication from both sender and receiver end.

Consequential effect of morphological asymmetry on courtship song and mating success has also been reported previously in birds, crickets, and grasshoppers. Male crickets and grasshoppers produce mating calls by stridulating specialized structures on forewings or tibiae of the hind legs (Figure 1d), and asymmetry in these organs alter the amplitude and structure of the mating call. Convincingly, female field cricket (*Gryllus campestris*) strongly prefer calls made by males with symmetrical sound resonators or harps (Simmons & Ritchie, 1996). In birds, asymmetric syrinx induces individual variations in vocalizations, but the link between FA and song attractiveness has remained speculative (Møller & Swaddle, 1997). In humans, facial FA and vocal attractiveness are negatively correlated, which was interpreted as a sign that a person's voice can reveal his/her genetic quality (Hill et al., 2017). Contrarily, other studies involving cricket frog *Acris crepitans* (Ryan et al., 1995) and *Drosophila montana* (Hoikkala et al., 1998) have found no link between asymmetry, acoustic cues and mate choice. Nonetheless, several examples of mating‐associated acoustic communications exist across taxa, where such links can be hypothesized and empirically tested. Below, we discuss two potential examples where this could be tested.

*In fishes*: acoustic communication is common among fishes, where males either produce low‐frequency sounds using bilateral drumming muscles attached to the swim bladder (Figure 1c), or high‐frequency sounds using bony attachments to pectoral fins and scraping their pharyngeal teeth (Amorim et al., 2015). Underwater, these low‐ and high‐frequency sounds travel longer and shorter distances, respectively, to attract females and advertise male quality (body size, body condition, and fat reserves) during courtship (Amorim et al., 2015). Although, several fish species choose their mates based on the information they infer from acoustic cues, it still remains untested if these acoustic cues play a role in FA‐mediated sexual selection. One could hypothesize that asymmetry in bilateral components of these acoustic organs (the drumming muscles, pectoral fins, and pharyngeal arch) produces abnormal mating calls that are ignored or disliked by the choosing individuals.

*In bats*: In echolocating bats, vocalization that is normally used for foraging also plays a role in sexual communication. Female horseshoe bat (*Rhinolophus mehelyi)* choose males with high‐frequency calls (a signal of body size) that are positively correlated with reproductive fitness (Puechmaille et al., 2014). Given that echolocation is in itself an epitome in acoustic communication, it is imperative to test if asymmetry of either the sender or receiver of such signals is detectable during sexual communication.

### 2.3 Vibrational communication (VC)

VC is another taxonomically widespread primitive mode of acoustic communication that is largely been ignored. Vibrations produced by striking body parts against surfaces communicate sexual interest, alarm, and other intraspecific information that induces complex social interactions in a range of animals from insects to elephants (Hill, 2008). VC is of three types: percussion or drumming where body parts are struck against a substrate; tremulation associated with oscillation, rocking, or jerking of body parts wherein vibrations are transmitted to the substrate via the legs; and substrate‐borne signals produced by tymbal or tymbal‐like mechanisms that scrape body parts against a substrate (Hill, 2008). VC is widely used in sexual communication across taxa to assess mate quality, yet we found no study that has tested whether asymmetry in bilateral VC‐generating organs affect signal quality or mate choice. Below, we discuss VC associated with mating in several species that can be used for empirical investigations.

#### 2.3.1 In invertebrates

The southern stink bug (*Nezara viridula*) is a model system for VC (Cocroft & Rodríguez, 2005), where individuals produces sex‐specific vibrational signals during courtship that propagates through plant stems, transmitting species‐, sex‐, and location‐specific information (Ota & Cokl, 2005). Male Australian crickets (*Balamara gydia*) court and attract females by tapping their abdomens on vegetation (Moore & Werner, 1989). Similarly, males of rasping crickets (*Gryllacrididae*) drum the substrate with tarsal hind limbs to which females respond in a duet (Field & Bailey, 1997). Female wolf spiders (*Hygrolycosa rubrofasciata*) recognize and choose males based on their foreleg drumming rate (Parri et al., 2002). Reviews on VC reflect how several insect species similarly utilize vibrations to locate, attract, assess, and/or choose mates (Hill, 2008); however, whether VC mediated by bilateral organs (legs, antennae, and tymbal) can signal mate asymmetry has never been investigated.

#### 2.3.2 In vertebrates

In fishes, male mottled sculpin (*Cottus bairdi*) produces ‘knock’ sounds by banging their head, the frequency of which in other species indicates male size and serves as a cue for mate choice (Whang & Janssen, 2004). The songbird blue‐capped cordon‐bleu (*Uraeginthus cyanocephalus*) performs complex courtship displays that can produce multimodal and multicomponent signals, including a tap dance‐like display wherein they rapidly stamp their feet several times to produce vibrations through the bird's perch (Ota, 2020).

VC is an archaic precursor to acoustic communication, and this is appreciable by understanding vibration sensing in anurans and reptiles. Extinct amphibians detected vibrations through their jaw in contact with the ground, and conduction through the quadrate bone of the jaw to the inner ear via bony tissue (reviewed in (Hill, 2008)). Caecilians, urodeles, some anurans, lizards, snakes, and amphisbaenians (worm lizards) lack tympanum and middle ear cavity but have stapes (a form of ear ossicle) attached to the shoulder girdle or skin that detects low‐frequency vibrations through their bodies, much of which is in direct contact with the substrate (Hill, 2008). Similar, but much larger ear ossicles are seen in mammals like elephants, horses, and seals, and facilitate acquiring information from bone‐conducted vibrations (Hill, 2008). The functioning of VC overlaps with auditory and mechanosensory perception, and this absence of distinctness could possibly explain its relative neglect. Nevertheless, acknowledging that VC has a role in sexual behavior opens ample opportunity to investigate if mate asymmetry can be signaled or perceived using vibrations.

### 2.4 Mechanosensory and tactile

Toward the culmination of courting, animals normally get physically intimate prior to copulation, allowing for mate assessment through tactile cues that plausibly continues even during copulation. These tactile behaviors can be simple males antennal tapping to more complex acrobatic moves. Male red‐sided garter snakes (*Thamnophis sirtalis*) compete to align themselves alongside a female's body and cloaca (Figure 1e), wherein the successful male entices the female by pressing his chin along the length of her body while continually attempting to intertwine his own tail with hers (Shine & Mason, 2001). Apart from the interlocking genitals of both sexes, males and females of many animals also have complementing morphological structures and mechanosensory organs that are bilateral and characteristically placed all over the body such that they interlock during mounting attempts and copulation. Such interlocking structures include hairs, spines, and clasping organs, malformations in which can affect mating success. Male cerambycidae beetles (*Stenurella melanura*) use tips of their long antennae to determine the abdominal tip of females while mating, and both sexes preferentially choose mates with symmetric antennae (MØller & Zamora‐muÑoz, 1997). Although not tested empirically for FA, similar issues with mating are known in other species. *Drosophila melanogaster* males that lack melanin in sex combs are impaired in their ability to grasp the female while mounting them (Massey et al., 2019), while males of other *Drosophila species* (*D*. *kikkawai*, *D*. *bipectinata*, and *D*. *ananassae*) with altered non‐intromittent genital spines fail to achieve copulation and have reduced pre‐ and post‐mating success (Grieshop & Polak, 2014; Polak & Rashed, 2010; Rodriguez‐Exposito et al., 2020).

The female genital tract of mammals and most other species is imbued with a rich ground plexus of autonomic nerves that regulate vascular and non‐vascular smooth muscle contractile activity, glandular secretions, nociception perception, immune cell interactions, and convey information to the central nervous system (CNS) regarding the internal environment and potential noxious stimuli to elicit appropriate behaviors (Brauer & Smith, 2015; Haesemeyer et al., 2009). This highly neurosensitive female organ could serve as a final aid in detecting male developmental instability by internally sensing differences in biomechanical stimuli from bilateral male genital adornments. The information thus gained can modulate female post‐copulatory mate discrimination through sperm utilization or expulsion. Although this mode of detecting FA warrants investigation, its plausibility is supported by the fact that (a) asymmetry in male genitals has been reported in diverse species including humans (Bogaert, 1997), and (b) male and female genital morphology and function have tightly coevolved to optimize fertilization success (Gredler et al., 2014; Simmons & Jones, 2007).

During copulation, females can assess cues on male quality from other modalities besides tactility that could then regulate copulation duration and other post‐copulatory decisions. Male flour beetles (*Tribolium*) display leg rubbing behavior during copulation that is speculated to provide tactile cues to females for post‐mating mate discrimination (Fedina & Lewis, 2008). Interestingly, these male beetles also have setiferous glands located on their prothoracic femora that produce sex pheromones, which could provide chemosensory cues during leg rubbing (Christian Olsson et al., 2006). The skin of anurans has certain sexually dimorphic and bilaterally located glands that secrete pheromones. Male frogs (*Ololygon centralis*) apply pheromones from inguinal glands directly to the female during amplexus, suggesting again that tactile and chemosensory cues can be communicated simultaneously (Brito et al., 2019). Such direct pheromone transfers from bilaterally located dermal glands during mating are also known in some salamander species (Houck, 2009). *Drosophila melanogaster* females seem to assess seminal fluid transferred by males during mating (Figure 1f) and if preferred, sing a copulation song by wing vibration in response, which in turn directly modulates male's ejaculate allocation in ways that prolong time to female remating (Kerwin & Philipsborn, 2020; Kerwin et al., 2020). Thus, copulation itself can provide multimodal cues that could reveal male asymmetry.

## 3 INDIRECT METHODS THAT ALLOW FEMALES TO ACCESS MATE SYMMETRY

Apart from discriminating against asymmetric males via cues signaled during courtship and copulation, females might also be able to do so even before being courted. Below, we review two phenomena, ‘extended phenotype’ (Schaedelin & Taborsky, 2009) and ‘mate‐choice copying’ (Vakirtzis, 2011), which have recently received immense attention in the field of sexual selection, and discuss if they could play a role in FA‐based mate choice. While the first method relies on assessing extended phenotypes built for sexual display by males, the second method involves copying choices of other females in an attempt to avoid erring during mate choice. Despite involving both visual and non‐visual cues, we found that these two indirect methods of mate choice have also been neglected in the FA sexual selection literature.

### 3.1 Extended phenotype

Many species manipulate their environments through genetically programmed construction behaviors for various purposes: webs spun by spiders for foraging; nests and burrows built by birds and rodents for roosting; and hives and mounds built by bees and termites for social living; and since these structures are phenotypes expressed beyond the body, they are termed ‘extended phenotypes’ (EP). In some species, these EPs are built by individuals of one sex (often males) solely to attract mates or as a part of their courtship ritual, functioning as a sexual trait, wherein the male's reproductive success is correlated with the quality of structures they build. These EPs have several benefits and drawbacks that are reviewed in Schaedelin and Taborsky (2009). Most importantly, they provide a proxy for male assessment in absentia, while on the other hand they are easily destroyed by rival males and can attract predators.

**FIGURE 2:**
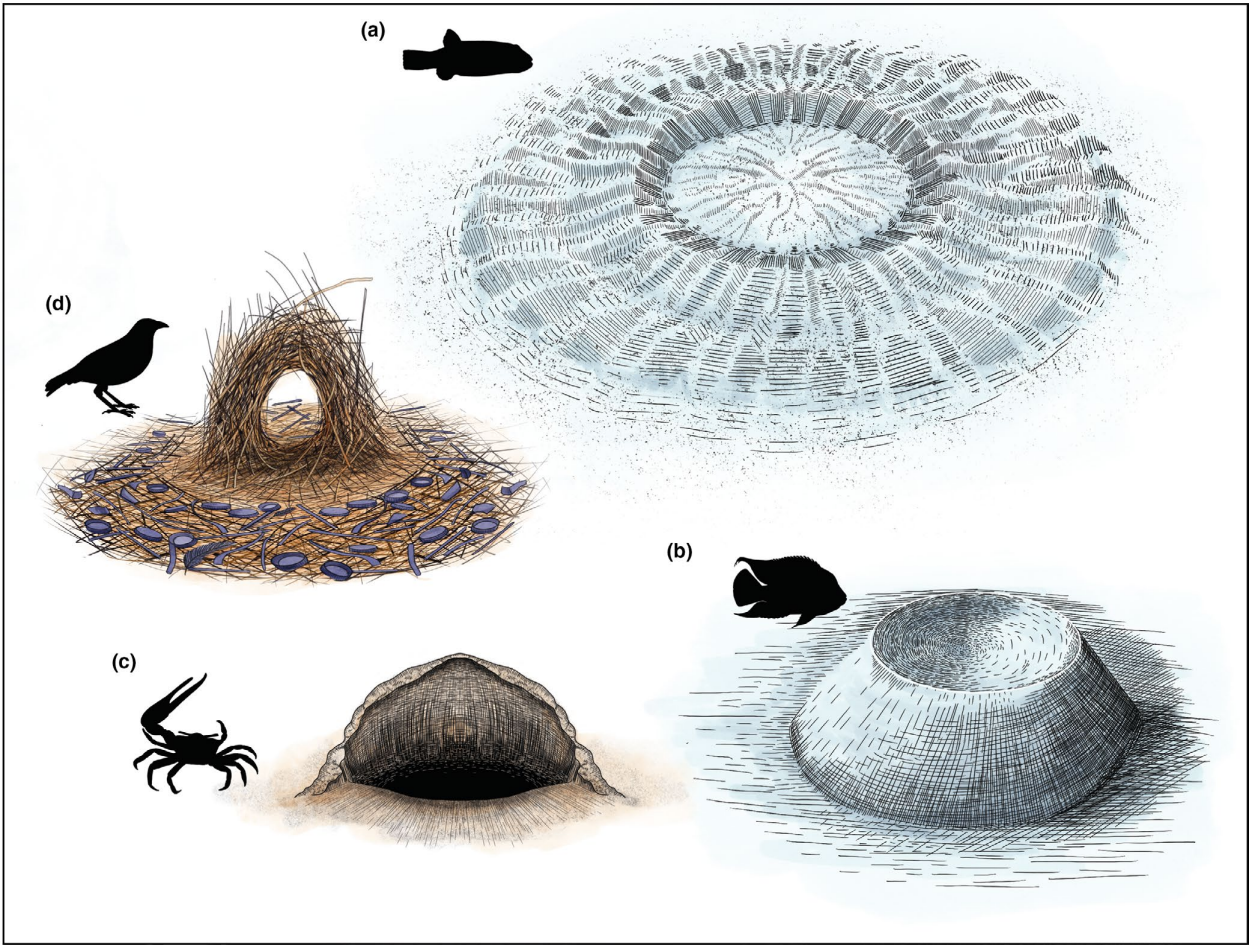
Extended phenotypes that could signal mate symmetry *(clockwise)*: a complex 2‐meter‐wide geometrically precise underwater nest built by the small Japanese pufferfish (a), a crater‐like nest built by a male cichlid fish on the seafloor (b), a hood‐like sand dune constructed by the fiddler crab male outside his burrow (c); and an elaborately decorated nest built by the Australian satin bowerbird male to attract a female (d)

Males across several species build overwhelming structures to grab a female's attention and these surpass their already rich multimodal courtship rituals. Males of diverse bird species build nests, bowers, and courting arenas decorated with objects like pebbles and petals, as courtship displays to lure females. Similarly, males of several fish species also indulge in building nests, craters, mounds, and sand patches to attract females during courtship (Barber, 2013; Barber et al., 2001) (Figure 2b). The most impressive of these structures is possibly the concentric geometric courtship arena (Figure 2a) built by a small male Japanese pufferfish (*Torquigener* sp.), wherein the male (12 cm long) constructs a complex arena that is 2 m in diameter, and takes over 7 to 9 days to complete (Kawase et al., 2013). Male ghost and fiddler crabs (subfamilies of *Ocypodinae*) build sand pyramids and sand dunes to attract females to their mating burrows (Figure 2c; reviewed in Schaedelin and Taborsky (2009)). Males of some spiders and insects (Diptera and Orthoptera) use silk, prey carcasses, burrows, leaf sculptures, and nuptial gifts to entice females while courting them (reviewed in Schaedelin and Taborsky (2009)). Responses of females in all the above examples stereotypically involve close inspection (both visually and tactilely) of these built structures prior to allowing or rejecting a male to court her, and studies suggest that females do infer male quality, size, and immune status as a correlate of the displayed structures (Schaedelin & Taborsky, 2009).

Even prior to the formulation of the DI‐SS hypothesis, female satin bower birds were shown to prefer bowers that were symmetrical (Figure 2d), and this structural symmetry of the bower was positively correlated with the mating success of the male (Borgia & Gore, 1986). Here, we speculate as to whether an individuals’ asymmetry can be reflected in structures they build, in ways that could mediate FA‐based mate choice. Such asymmetry in extended phenotypes could possibly result in two ways: (a) morphological asymmetry in organs (asymmetrical tools) used in construction; and (b) asymmetry in the builder's sensory modules (asymmetric perception) that is required to execute the construction. Since most extended phenotypes are larger than the male body size, they possibly function as amplifiers that could magnify the level of asymmetry proportionally. Conversely, where the size of the builder and the structure are similar, females inspect these structure by physically testing them (crawling through loops or tunnel openings), wherein the ease of such maneuvers for females could indicate the size and symmetry of the potential male (Barber et al., 2001). These examples suggest that EPs are yet another wealthy source of cues that could indirectly signal asymmetry of potential mates.

### 3.2 Mate choice copying

Mate choice copying is yet another phenomenon that is widespread across taxa, wherein the likelihood of choosing a mate is influenced by the apparent choice of other more experienced conspecifics (Vakirtzis, 2011). Although both males and females indulge in mate choice copying, this behavior is more rampant among females in many species. While most studies report how females copy conspecific preference (acceptance) of a male, the definition of mate choice copying per se also allows females to similarly copy male rejection, but this latter aspect has seldom been tested (Vakirtzis, 2011; Witte & Ueding, 2003). A meta‐analysis of mate choice copying literature revealed that virgin rather than mated females were more likely to copy mate choices of other conspecific females, whose relative age was irrelevant. Nevertheless, naive female guppies (*Poecilia reticulata*) show preference to copy mate choice of older females (Dugatkin & Godin, 1992). This mate choice copying behavior is speculated to have evolved as a cost avoidance or shortcut strategy, wherein the costs are time and energy associated with active mate choice. Alternatively, it has been proposed that mate choice copying evolved to facilitate mate discrimination by reducing the uncertainty or error component in mate assessment process (reviewed in Vakirtzis, 2011). Thus, individuals using such information (sexual or non‐sexual) gleaned from observing conspecifics (Hoppitt & Laland, 2008) can benefit from reduced decision time during mate choice and assessing competing choices (Giraldeau et al., 2002).

Here, we hypothesize that mate choice copying (mate acceptance or rejection) is yet another indirect way through which asymmetric individuals may be discriminated during courtship. Individuals indulging in mate choice copying tactically overcome their inexperience in mate choice or mitigate the costs of such assessments, but can indirectly bias their choice for symmetric mates if the model had preferred a symmetric mate. The ability to assess potential mate quality increases with age as a consequence of learning, so mate choice copying by naive individuals might set a benchmark on quality of preferable mates for future mating's. Importantly, its known that females copy not only other females’ preferences for certain males but can generalize these preferences to other males with similar traits (reviewed in Vakirtzis (2011)). Furthermore, mate choice copying could benefit males too: courting and copulation with a model female is likely to reduce courtship effort during subsequent mating with the onlookers, possibly explaining why males indulge in elaborate, multimodal, and costly courtship rituals that often seem to be exquisitely orchestrated toward a single individual.

Mate choice copying is largely thought to be mediated by visual observations, but if non‐visual modalities are utilized this could bypass the necessity that copying female must share spatiotemporal proximity to third‐party copulations. Female Norway rats (*Rattus norvegicus*) and female mice (*Mus musculus*) prefer mating with recently copulated males that they detect using chemosensory cues (Galef et al., 2008; Kavaliers et al., 2006). Acoustics‐mediated mate choice copying is known among brown‐headed cow‐birds (*Molothrus ater*) (Freed‐Brown & White, 2009), wherein the characteristic chatter sound emitted by females when successfully courted by males signals to copying females an assessment of male's quality. Thus, irrespective of whether mate choice copying is mediated through visual or non‐visual cues (Vakirtzis, 2011), this behavior could indirectly facilitate sexual selection for symmetric mates, warranting further research.

## 4 CONCLUSION

Researchers investigating developmental instability suffer immense dilemma while measuring, analyzing, and interpreting data on FA in a sexual context. The problem begins with choosing a ‘relevant’ bilateral trait, then accurately measuring FA (FA is extremely small, i.e., 1–2% of trait size, and have statistical properties similar to those generated from errors during measurement) (Polak, 2003), and finally interpreting the data. This dilemma possibly explains the extensive literature on ‘how to’, ‘what to’, and ‘what not to’ do while planning, analyzing, and interpreting data on FA (Dongen, 2006; Palmer & Strobeck, 1986, 2003). Yet, additional layers of concern emerge when FA is studied in the context of sexual selection (Rohde et al., 1997; Uetz & Taylor, 2003). These include (i) overall reduced quality of individuals owing to the methods used to induce FA (e.g., environmental or genetic stress during development); (ii) assessment of low levels of FA in mates amid background noise (i.e., low signal‐to‐noise ratios); (iii) difficulty to differentiate if mate preference was affected by FA or other traits that are correlated with FA (e.g., surgical manipulation of tail feather symmetry in birds would invariably affect their flight performance); and (iv) issues pertaining to suitable controls, repeatability, and intraindividual variations.

The FA‐related challenges listed above might not be unique to researchers, it is likely that individuals across taxa face the same dilemma in assessing asymmetry during mate choice. However, the multimodal cues and the indirect strategies that could signal individual asymmetry we review here present unique evolutionary solutions that diverse species across taxa have evolved in response to certain aspects of this dilemma over millions of years. For example, many of these cues can easily amplify the low levels of morphological asymmetry and thus help in its detection. In our recent study, female *Drosophila* were shown to discriminate against males with low levels of wing asymmetry through the consequent asymmetry in male courtship song features (Vijendravarma et al., 2022). Interestingly, ability to compare the vibration alternatively generated by the two male wings is an efficient way to amplify a very low morphological asymmetry. The study also overrides several experimental caveats that former studies were criticized for. These include: (a) FA in males was induced by rearing *Drosophila* under altered gravity, wherein despite being asymmetric the reproductive fitness of males was not affected; (b) female preference was assayed under different mate choice paradigms; (c) males that were competed in the mate choice assays were randomly chosen from the same rearing condition; (d) FA level in males that were successful or unsuccessful in securing a mating was determined post‐female choice; (e) manipulation of different female sensory modalities was used to determine the sensory basis of female preference for male symmetry; and (f) genetic and surgical manipulation of male symmetry was used as additional FA‐inducing methods to corroborate the findings (Vijendravarma et al., 2022).

In conclusion, although simple, the fundamental idea that individuals can improve their fitness by biasing their choice toward mates with higher developmental stability appeals to species across taxa. This possibly explains why the developmental instability–sexual selection hypothesis is still debated, despite the immense equivocal literature generated as a consequence. Those supporting the hypothesis need to reconsider that aspects of the hypothesis warrants refinement while those against it must acknowledge the short comings in the empirical data that refute the hypothesis. This review highlights the layers of complexity underlying the superficial simplicity of this hypothesis: the diversity of the multimodal non‐visual cues that could signal asymmetry during courtship and the indirect ways in which mate asymmetry can be assessed. Furthermore, the dynamic exchange of information occurring between the sexes or the decision algorithms animals use to process this information are not even considered as a part of this complexity. Thus, the importance of evoking the ecological, behavioral, and evolutionary aspects of animals while designing experiments and interpreting empirical data that test the DI‐SS hypothesis cannot be overemphasized.

## CONFLICT OF INTEREST

Both RKV and PL declare that they have no conflict of interest.

## AUTHOR CONTRIBUTIONS

**Roshan Kumar Vijendravarma:** Conceptualization (lead); Funding acquisition (equal); Methodology (equal); Project administration (lead); Resources (equal); Validation (lead); Visualization (lead); Writing – original draft (lead); Writing – review & editing (lead). **Pierre Leopold:** Conceptualization (supporting); Funding acquisition (equal); Project administration (supporting); Resources (equal); Writing – original draft (supporting); Writing – review & editing (supporting).

## Data Availability

This article is a literature review that does not include any reportable data.
